# Small RNA-Sequencing: Approaches and Considerations for miRNA Analysis

**DOI:** 10.3390/diagnostics11060964

**Published:** 2021-05-27

**Authors:** Sarka Benesova, Mikael Kubista, Lukas Valihrach

**Affiliations:** 1 Laboratory of Gene Expression, Institute of Biotechnology, CAS, BIOCEV, 252 50 Vestec, Czech Republic; sarka.benesova@ibt.cas.cz (S.B.); mikael.kubista@tataa.com (M.K.); 2 Laboratory of Informatics and Chemistry, Faculty of Chemical Technology, University of Chemistry and Technology, 166 28 Prague, Czech Republic; 3 TATAA Biocenter AB, 411 03 Gothenburg, Sweden

**Keywords:** small RNA-seq, miRNA, diagnostics

## Abstract

MicroRNAs (miRNAs) are a class of small RNA molecules that have an important regulatory role in multiple physiological and pathological processes. Their disease-specific profiles and presence in biofluids are properties that enable miRNAs to be employed as non-invasive biomarkers. In the past decades, several methods have been developed for miRNA analysis, including small RNA sequencing (RNA-seq). Small RNA-seq enables genome-wide profiling and analysis of known, as well as novel, miRNA variants. Moreover, its high sensitivity allows for profiling of low input samples such as liquid biopsies, which have now found applications in diagnostics and prognostics. Still, due to technical bias and the limited ability to capture the true miRNA representation, its potential remains unfulfilled. The introduction of many new small RNA-seq approaches that tried to minimize this bias, has led to the existence of the many small RNA-seq protocols seen today. Here, we review all current approaches to cDNA library construction used during the small RNA-seq workflow, with particular focus on their implementation in commercially available protocols. We provide an overview of each protocol and discuss their applicability. We also review recent benchmarking studies comparing each protocol’s performance and summarize the major conclusions that can be gathered from their usage. The result documents variable performance of the protocols and highlights their different applications in miRNA research. Taken together, our review provides a comprehensive overview of all the current small RNA-seq approaches, summarizes their strengths and weaknesses, and provides guidelines for their applications in miRNA research.

## 1. Introduction

MicroRNAs (miRNAs) represent a class of short (~22 nucleotides) non-coding RNA molecules that are well conserved across various species. They are known mainly for their post-transcriptional regulation of gene expression via degradation and translational repression [1]. However, there is a growing body of evidence that they can also act as an expression activator upon specific conditions such as cell cycle arrest [2,3,4]. Moreover, the localization of mature miRNA formed in the nucleus has led to the discovery of their ability to regulate gene expression even at the transcriptional level [5].

MiRNA genes are located both within defined transcription units as well as in intergenic regions, where they create clusters transcribed as polycistronic RNA [6]. Their biogenesis involves transcription by RNA polymerase II resulting in structured primary miRNA (pri-miRNA), which is processed by the Microprocessor complex into a precursor miRNA (pre-miRNA). Pre-miRNA is subsequently exported to the cytoplasm and cleaved by the Dicer protein complex, creating the duplex miRNA. One strand of miRNA duplex is loaded into the Argonaut (AGO) protein, creating the RNA-induced silencing complex (RISC), which then binds to the mRNA using the complementary seed sequence of the loaded mature miRNA [7]. Alternative cleavage by the Microprocessor complex and the dicer leads to the production of isomirs, biologically active miRNA isoforms with alternative 3′- and/or 5′-end compared to the canonical mature sequence [8].

Since the discovery of the first miRNA lin-4 in nematodes, more than 38,000 miRNAs have been detected in 271 organisms, including 1917 human miRNAs (miRBase v22) [9]. It has been proposed that more than 60% of human protein-coding genes can be directly post-transcriptionally regulated by miRNAs [10]. As a single miRNA can regulate up to hundreds of genes, miRNAs are supposed to act as hub genes in regulatory networks and are thought to play a role in almost all biological processes. In the field of cancer research, miRNAs became the subject of numerous studies, which demonstrated their active role in the disease and showed cancer-specific gene expression [11,12,13,14]. The presence of cancer-specific miRNA profiles, together with the presence and stability of miRNAs in various biofluids have made them useful biomarkers allowing for non-invasive cancer diagnosis and prognosis [15,16].

Microarrays, reverse transcription-quantitative PCR (RT-qPCR) and RNA-sequencing (RNA-seq) are the three most common approaches for miRNA profiling. For a long time, microarray analysis represented the most commonly used high-throughput technology for miRNA profiling [12]. However, this hybridization-based technique does not allow for absolute quantification, identification of novel miRNAs, and separate detection of canonical miRNAs and their isomiRs, of which the importance has only started to be recognized recently [17,18,19]. Moreover, microarray-based methods usually require high quantities of input RNA. This may be a limiting factor for the non-invasive analysis of liquid biopsies, where the concentration of miRNAs is rather low [20]. Compared to the microarray, RT-qPCR allows for absolute quantification, has a broad dynamic range, high sensitivity and allows for the analysis of low RNA inputs [21]. For its non-demanding instrumentation and availability of expertise in many laboratories, RT-qPCR stands out as a gold standard method for targeted miRNA analysis. RNA-seq represents the last major approach for miRNA analysis. The main advantage of small RNA-seq is its non-targeted character, allowing for simultaneous detection of novel miRNAs, detection of isomiRs as well as other small RNA species. The disadvantages are represented by the complex experimental workflow which introduces various types of biases and demanding computational analysis. To minimize these disadvantages, there have been substantial technological developments within the past five years resulting in the introduction of several new small RNA-seq protocols (Table 1). However, a comprehensive overview and comparison of all the methods, which would serve as a guideline for new users, is missing. Although several comparative studies testing the performance of the new small RNA-seq protocols have appeared in recent years, they have not been properly reviewed yet. To fill the gap, we summarized the recent knowledge on new approaches for small RNA-seq analysis as well as reviewed all recent comparative studies. Here, we aim to provide a comprehensive overview of the small RNA-seq technology that will facilitate to any new user the ability to make an informed selection of the best-suited protocol for a particular experimental question. We hope that our efforts will promote the standardization of the technology and its broader applications in the near future.

## 2. Small RNA-seq Technology Overview

RNA-seq has undoubtedly revolutionized the characterization of the small transcriptome, enabling high-throughput profiling and discovery of novel forms of short non-coding RNAs (miRNAs, piRNAs, tRNAs, siRNAs, snoRNA, etc.) [27]. The first application was demonstrated in 2005, when small RNA-seq was used to profile various types of small RNAs of *Arabidopsis thaliana* [28]. A year later, small RNA-seq identified 18 new miRNAs in *Caenorhabditis elegans* [29]. Since then, there have been more than 700 human miRNA expression profiling studies, which used small RNA-seq and were deposited on Gene Expression Omnibus (GEO) [30]. With the development of single-cell RNA-seq, it is now possible to analyze miRNAs even in individual cells [31].

The small RNA-seq workflow involves three main steps: (i) isolation of RNA; (ii) cDNA library construction; and (iii) sequencing [32]. Small RNA may be isolated using conventional total RNA extraction methods followed by length separation or binding of small RNAs to specific proteins. As small RNA enrichment may lead to losses [33] and/or isolation-specific biases [34,35], analysis of total RNA is an alternative and usually preferred strategy. For example, recent studies reported a higher proportion of reads mapping to small RNA/miRNAs and a higher number of detected miRNAs with protocol isolating total RNA from human plasma than with small RNA enrichment protocol [34,36]. The library construction starts with reverse transcription (RT) of small RNAs, producing complementary DNA (cDNA) for amplification and sequencing. As miRNA molecules are of short length, they are extended by ligation or polyadenylation, which introduce primer-binding sites for RT and subsequent amplification. The extension step, especially the ligation, is considered as the most prominent source of bias in small RNA-seq [32,37,38]. Different affinity of adaptors to target molecules causes artificial changes in the true small RNA abundances, which confounds the analysis. The amplification step of cDNA molecules represents another source of bias, which originates from the differing efficiencies of the PCR while amplifying molecules of different lengths and secondary structures [39]. This leads again to artificial shifts in the detected signals. This PCR bias is well known in the field of mRNA sequencing, and is usually mitigated through the use of unique molecular identifiers (UMIs) [40,41]. UMIs allow for the distinguishing of reads generated from an identical molecule that was amplified by PCR. The last step in the small RNA-seq workflow is sequencing. Although this step might be associated with certain types of biases (lane-, flow-cell effects), these are mostly considered as minor ones having a subtle effect on the overall bias level [42].

During the past years, the major technical development in the small RNA-seq was focused on the reduction of the ligation and PCR bias. At present, there are 15 commercial protocols available, which cover three distinct approaches: (i) original two-adaptor ligation; (ii) improved two-adaptor ligation; and (iii) ligation-free (polyadenylation-based) approach (Table 1). Besides these methods, there is a group of alternative technologies relying on the hybridization of miRNAs to target probes that completely avoid the ligation or polyadenylation step and in some cases also the PCR step and NGS readout. These techniques usually offer a simplified workflow that is suitable for routine and automatized analysis. On the other hand, they are limited in the spectrum of analyzable miRNAs (given by the set of predesigned probes), which limits their discovery potential.

### 2.1. Two-Adaptor Ligation-Based Methods

**Figure 1:**
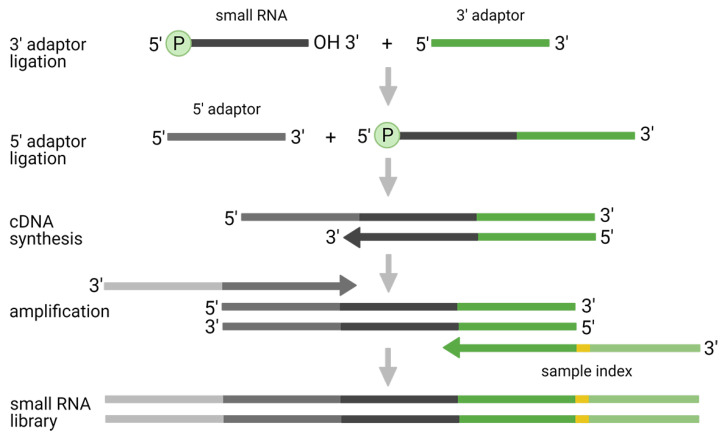
Original two-adaptor ligation protocol for small RNA-seq analysis.

The traditional two-adaptor ligation approach utilizes the ligation of the pre-adenylated 3′-end adaptor followed by the ligation of the 5′-end adaptor (Figure 1). Although this approach has been successfully applied in the majority of small RNA-seq studies (for historical reasons), its application is associated with several issues, including: (i) ligation bias; (ii) preferences to certain small RNA classes; and (iii) formation of adaptor dimers.

First, the ligation reaction is a major source of bias that negatively affects miRNA quantification in traditional two-adaptor small RNA-seq protocols [37,43,44]. Consequently, measured profiles do not reflect true miRNA abundances, the majority of miRNAs are systematically over- or under-estimated and some may be completely undetected. The primary cause of this phenomenon was shown to be secondary structures of both ligation substrates, i.e., miRNAs and adaptors [38,45]. The secondary structure of the adaptor defines the preference to certain miRNAs and vice versa. This results in some miRNAs ligating with high efficiency whereas others with substantially lower, leading to bias in the data. The issue might be diminished by the application of the improved two-adaptor ligation methods (see next chapter). Apart from that, there is also a possibility for in silico correction of measured expression values [46]. The model uses the calculation of bias factor, which is learned for each miRNA on an equimolar pool of synthetic miRNAs and is subsequently applied for the correction of real data. Although promising, the correction is problematic for low input samples where few miRNAs consume most of the reads. This can result in some of the low abundant miRNAs being completely missed (dropouts) with the inability for correction.

Secondly, as the method utilizes T4 RNA ligases that recognize the hydroxyl group on the 3′-end and the phosphate group on the 5′-end of miRNAs, it enables profiling of all RNA species with such modifications. This includes long non-coding RNAs, which need to be removed by bead-based size selection or polyacrylamide gel electrophoresis (PAGE) purification. Other small RNAs with ligase-compatible 5′- and 3′-end modifications and similar lengths, such as piRNAs, are retained in the library and analyzed [42], whereas others with ligase-incompatible modification will be missing. For example, small RNAs with trimethyl-guanosine cap on 5′-end such as small nuclear RNAs (snRNAs) are not captured and sequenced. For profiling of these missing small RNA classes, there are protocols such as ScriptMinerTM (Cambio Ltd.) utilizing enzymes, which degrade caps and polyphosphate groups on the 5′-end and make these molecules analyzable [23,47]. Another approach to capture small RNAs regardless of their 5′-end modifications is to add the 5′-adaptor after RT and target the cDNA molecules [48,49]. Lastly, the recently-introduced phospho-RNA-seq method utilizes T4-polynucleotide kinase, which is used for phosphorylation and dephosphorylation in order to capture small RNAs regardless of their end modifications [50]. Focused on 3′-end, utilization of T4 Rnl2tr ligase in optimized conditions improves capturing of small RNA species with 2′-O-methyl on 3′-end [51].

The final problematic aspect of the traditional two-adaptor approach is the formation of adaptor dimers, which may consume a substantial proportion of the sequenced reads when not removed properly. Especially in low input samples, where libraries are prepared using a higher number of PCR cyclers, this may cause complete loss of lowly abundant small RNAs. There are multiple approaches how to remove or limit the formation of adaptor dimers. These include: (i) bead-based or PAGE removal of adaptors from libraries [52]; (ii) enzymatic removal of 3′-adaptor excess limiting the formation of dimers [23,47]; (iii) usage of chemically-modified adaptors which do not ligate without insert [22]; (iv) annealing of RT primer in equimolar concentration to 3′-adaptor before ligation of 5′-end adaptor which blocks RT of dimers [53]; (v) blocking RT of adaptor dimers by adding oligonucleotide with complementary sequence [54]; (vi) and usage of CRISPR/Cas9 carrying sgRNAs specific to adaptor dimers which leads to their selective depletion [55]. The last approach can be used also for the removal of highly abundant miRNAs, which would consume a large proportion of the reads, but are not of interest. An alternative method for the removal of highly abundant miRNAs is to block them as a substrate for ligase by the addition of oligonucleotide complementary to the undesired miRNA [56,57]. Some of the aforementioned approaches to prevent adaptor-dimers are a part of commercially available ligation-based protocols, including the original two-adaptor ligation-based (Table 1). Moreover, there are other improvements such as PEG addition, specific adaptor concentrations, and specific ligation temperature, which increase the yield of ligation and may contribute to the mitigation of overall bias [22].

### 2.2. Improved Two-Adaptor Ligation-Based Methods

**Figure 2:**
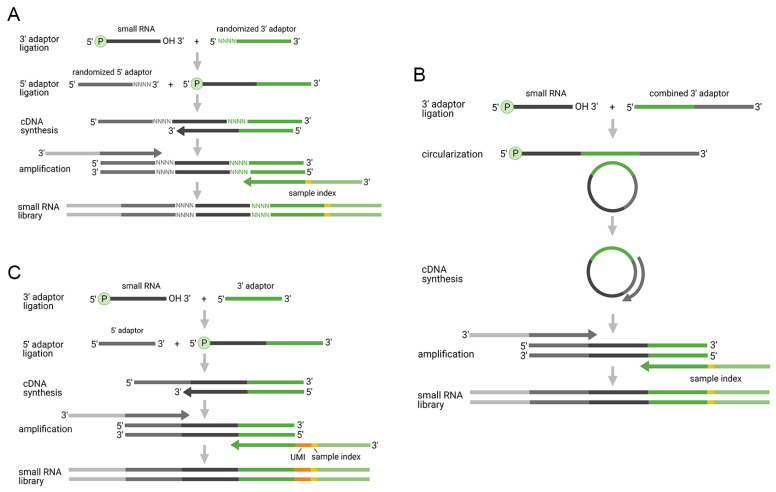
Improved two-adaptor ligation-based methods for small RNA-seq analysis. The reduction of ligation or PCR bias is achieved by: (**A**) randomized adaptors; (**B**) single adaptor-ligation and circularization; and (**C**) UMIs.

The improved two-adaptor ligation approaches aim to prevent or mitigate the ligation or PCR bias. Currently, there are three approaches available: (i) ligation of two adaptors with randomized nucleotides (Figure 2A); (ii) ligation of single adaptor and subsequent circularization (Figure 2B); and (iii) ligation of two adaptors with UMIs (Figure 2C).

The superior performance of methods utilizing randomized adaptors over the traditional two-adaptor approach is frequently reported in the literature [58,59,60], including our own data [61]. Currently, there is only a single commercially-available protocol—NEXTflex provided by PerkinElmer. However, the superior performance of the randomized-adaptor approach was reported using an in-house protocol as well [50]. The application of adaptors that contain a random oligonucleotide part was originally proposed to study the cause of ligation bias on the 3′-end of miRNAs [62]. The study showed that ligases do not prefer any miRNAs according to their primary sequence, but they are rather biased by secondary structures presented in the adaptors and targeted small RNAs. Thus, the inclusion of random parts in the adaptors, efficiently decreased ligation bias. Similarly, the usage of “High Definition” (HD) adaptors containing four random bases in both adaptors led to a substantial decrease of ligation bias [44]. Of note, a subsequent study complemented the data and demonstrated that the inclusion of randomized parts diminished the ligation bias regardless of the distance to ligation junction [38]. The finding that miRNAs preferentially ligate to the adaptors which increase the potential to create secondary structures motivated the development of a new approach for optimal adaptor design [38]. Using this model, adaptors are designed to include randomized oligonucleotides and complementary regions between 5′- and 3′-adaptors, which induce structures preferred by ligase. Lastly, a recent study showed that small RNA capture efficiency can be further improved by a combination of randomized adaptors with the addition of nucleotide modifications and optimized adaptor concentration [63]. Noteworthy, randomized nucleotides can be used as UMIs as well and mitigate the PCR bias, as has been demonstrated in a recent study [60]. The utilization of 4 + 4 random nucleotides from both 3′- and 5′-end adaptors led to a substantial decrease of PCR bias, which was estimated using an equimolar pool of 962 miRNAs. However, the experimental design did not account for the total number of molecules and the limiting number of unique combinations given by 4 + 4 nucleotides (4^8^), which caused over-deduplication and over-estimation of PCR bias [64]. Therefore, the use of randomized adaptors as UMIs should be made with caution and should be considered with respect to the concentration of the ligated RNAs, especially those presented in high copy numbers.

The ligation of a single 3′-end adaptor and subsequent circularization represents another promising approach that has been recently introduced to mitigate ligation bias [25]. The decrease of ligation bias is primarily achieved by intramolecular circularization, which is more efficient than intermolecular 5′-ligation. To prevent self-circularization of the 3′-adaptor, its 3′-end is blocked by a phosphate group. After successful ligation of the 3′-adaptor, the miRNA-adaptor construct is purified, dephosphorylated, circularized and RT is performed on the circularized molecules. Although the system is well designed to mitigate 5′-end bias, 3′-end bias is supposed to be still present. This is reflected by contradictory results in the literature. Whereas the original and recent benchmarking studies showed less ligation bias than the protocol based on randomized adaptors [25,65], ours and others data showed rather average to poor perfomance [61,66]. However, the contradictory results were achieved by different chemistries. The positive data were reported using the Somagenics RealSeq-AC Kit intended for tissue samples, whereas later two further reports used Somagenics RealSeq-biofluids Kit or SMARTer microRNA-Seq Kit from Takara Bio. Further assessment of protocol-specific parameters and their impact on the level of ligation bias is therefore needed to provide further insight.

The last group of methods focused on the mitigation of PCR bias. This is achieved via the utilization of UMIs that may be introduced in one or both adaptor sequences, alternatively in the RT primer. There is an ongoing debate on the importance of PCR bias. Where early reports highlighted a minor role of PCR bias [38,43,67], recent studies called this in question [41,60]. The amount of starting material and sequencing depth was shown to influence PCR duplicates frequency and their removal increased reproducibility of the data [41]. Currently, two-adaptor ligation protocols with UMIs are commercially available from Qiagen and GenXPro [68]. The QIAseq miRNA Library Kit from Qiagen includes adaptors containing 12bp UMIs, which produce enough unique sequences even for high abundant miRNAs, therefore, deduplication does not result in their under-estimation. In comparison, the protocol performs worse than approaches using randomized adaptors, but better than original two-adaptor ligation protocols [65,66]. Our own recent study showed a similar outcome, scoring the QIAseq protocol close to other ligation mitigating approaches, suggesting that also other protocol-specific factors might play a role in its improved performance [61].

### 2.3. Ligation Free Methods

**Figure 3:**
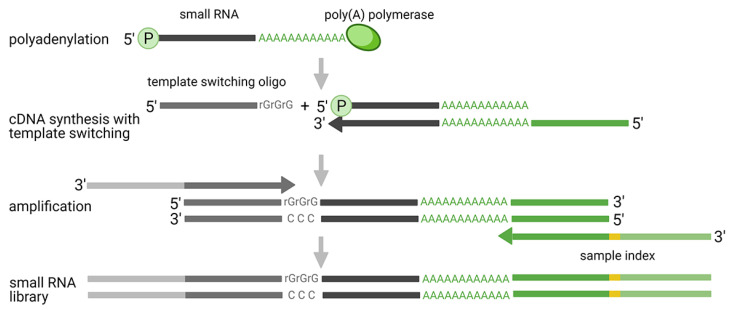
Polyadenylation and template switching mechanism applied in small RNA-seq analysis.

Ligation-free methods include protocols based on polyadenylation with template switching (Figure 3) and probe-based techniques, which enable targeted profiling of known miRNAs. Although the second group of methods does not rely, in all cases, on RNA-seq, they were included in this chapter as they represent an interesting alternative to traditional small RNA-seq. They offer a simplified workflow and computational analysis, while bypassing the biases associated with the ligation and PCR step.

Historically, the polyadenylation method was introduced together with the original two-adaptor ligation approach and contributed to the discovery of various mammalian miRNAs [69]. The first protocol employed polyadenylation of the 3′-end and subsequent ligation of the 5′-end adaptor. Nowadays, the 5′-ligation was replaced by the addition of the 5′-adaptor using template-switching activity of reverse transcriptase (RTase), a mechanism originally introduced for full-length reverse transcription of mRNAs [70]. This technology uses Moloney murine leukemia virus (MMLV) RTase, which adds non-templated cytidines at the 5′-end of each cDNA molecule. The added stretch of nucleotides consequently serves as a binding site for 5′-end adaptor. Currently, there are two companies providing this type of solution for small RNA analysis: Diagenode and Takara Bio. The major advantage of these protocols is the independence on ligation, which ensures more accurate miRNAs quantification compared to ligation approaches. However, there are also disadvantages, which need to be considered. In recent comparative studies, both of the protocols showed that a high proportion of reads did not align to miRNA sequences, but to other small RNA classes, and also that a high proportion of reads did not map at all [65,66]. Consequently, the low miRNA mapping rate might increase the sequencing costs (to achieve the desired coverage of miRNAs) or result in an insufficient number of miRNA reads, thereby limiting further analysis. On the other hand, the signals from other small RNA classes may provide extra information and lead to new discoveries. Another aspect for consideration is that polyadenylation does not add a specific number of adenosines to the template. Therefore, it is not possible to distinguish native nucleotides from those artificially added and determine the exact 3′-end of sequenced miRNAs. Similarly, the template switching activity of RTase is supposed to add three non-templated cytidines. However, the exact number may vary, which complicates recognition of the 5′-end of miRNAs. These biases cause problems during the mapping of sequencing reads to the reference, which results in a high proportion of discarded reads. Moreover, the problems with recognition of exact 5′- and 3′-end impair quantification of isomiRs and other closely related molecules.

The application of small RNA-seq methods is especially advantageous where novel forms of small RNA/miRNAs are of interest. However, these methods suffer from protocol-specific biases that do not allow for the direct cross-study comparison and complicate meta-analysis. Moreover, the complex workflow negatively affects their applicability for clinical diagnostics where rapid turnaround time and robust protocols are desirable. To answer these requests, several modern hybridization methods have been recently developed, including Nanostring nCounter, Abcam FirePlex and EdgeSeq from HTG Molecular Diagnostics. Nanostring nCounter utilizes direct molecular barcoding and digital detection of targeted molecules by color-coded probes [26,71]. Unique oligonucleotide tags (miRtags) are ligated to the 3′-end of targeted miRNA, whereas the specificity of the reaction is ensured by bridge oligonucleotide with complementary sequence to the targeted miRNA and miRtag. Created complexes are immobilized and after several washing steps, color-coded barcodes are counted. Each barcode corresponds to a specific miRNA target, which allows its reliable identification. Currently, the technology allows for the quantification of over 800 highly curated human, mouse and rat miRNAs. FirePlex miRNA assay from Abcam leverages the hydrogel-based microparticle technology [72]. First, miRNAs are bounded to the central miRNA-specific region of hydrogel particles, which contain two end regions with different fluorescent intensities serving as barcodes. Bounded miRNAs are ligated to universal adaptors and eluted from hydrogel particles. Subsequently, miRNAs are amplified using biotinylated primers complementary to the adaptors. After amplification, biotinylated miRNA targets are re-hybridized to hydrogel particles and a flow cytometer is used for detection of reporters specific to biotin. This method allows for quantification of 65 miRNAs in a single reaction, different disease-specific panels are available, and the workflow enables analysis of crude biofluid samples, eliminating bias introduced by isolation [34,35,73]. The analysis of crude biofluid samples is also possible with EdgeSeq from HTG Molecular Diagnostics. The protocol utilizes hybridization probes designed for specific binding of 2083 miRNAs annotated in the miRBase v20 database. After hybridization, unbound RNA and probes are digested by S1 nuclease. Targeted miRNAs are further released from capturing probes, these are amplified and sequenced using Illumina sequencing [74]. The complete process of library preparation is automatized, which saves hands-on time and eliminates errors. Moreover, the computational analysis is standardized leading to reliable results that are comparable across laboratories.

## 3. Benchmarking Studies

Over the past five years, several new approaches for small RNA profiling have been introduced and the majority of them transformed into commercial products (Table 1). This naturally raised an interest to compare their performance in various experimental settings, using different types of samples and evaluation metrics. The tested samples include plant or human total RNA, RNA isolated from immune cells or cell cultures, human serum and plasma samples for their frequent use in diagnostics, and lastly equimolar mixture of artificial miRNAs (miRXplore) allowing quantification of the ligation and PCR bias. The basic parameters, which were evaluated in most of the benchmarking studies, include: (i) yield—the proportion of mapped reads; (ii) reproducibility—variation of technical replicates; (iii) specificity—ability to detect only true targeted sequences; (iv) sensitivity—ability to detect targeted sequences considering given sequencing depth and minimal number of targeted miRNA reads; (v) accuracy—proportion of over- or under-estimated sequences; and (vi) ability to detect differentially expressed RNAs. Additionally, protocols were evaluated in terms of the workflow complexity (hands-on time, number of pipetting steps, robustness), ability to analyze low RNA input samples, level of multiplexing, and total costs.

We conducted a thorough literature search and identified twelve comparative studies that compared at least two different approaches for small RNA-analysis. The results are summarized in Table 2, alongside major conclusions. Only three studies, including our own, compared all available approaches for small RNA-seq [25,61,65]. In the study of Barberan-Soler et al. [25], the authors introduced the circularization approach and compared it with remaining protocols. The circularization protocol outperformed others in most of the tested metrics. It greatly reduced ligation bias and enabled the identification of the largest variety of miRNAs found in human brain RNA. The reduced bias allowed also for a robust miRNA quantification regardless of RNA input levels. The study of Herbert et al. [65] confirmed the result from the previous study, demonstrating a high accuracy and sensitivity of the circularization approach. The comparison was conducted on total RNA isolated from mesenchymal stem cells and miRXplore and contained all major RNA-seq approaches. Of note, both of the studies used an early-release beta version of the Somagenics RealSeq-AC protocol. In our recent study, we used Somagenics RealSeq-Biofluids protocol and observed a rather moderate reduction of ligation bias [61]. The non-optimal performance of another circularization protocol SMARTer microRNA-Seq Kit (Takara Bio) was reported by the study of Heinicke et al. [66], where the majority of reads were lost during the mapping procedure. Together, the results showed that other protocols, or sample-specific factors, might play a role and therefore require further investigation. The study of Herbert et al. [65] included also the hybridization-based approach nCounter (NanoString) and showed its low correlation with other RNA-seq based methods. This is in accordance with a recent study comparing RNA-seq with all modern hybridization platforms, demonstrating substantial protocol-specific biases limiting inter-platform comparison [75]. Lastly, our recent study compared all available RNA-seq approaches for miRNA analysis in human plasma, including the hybridization-based EdgeSeq protocol from HTG Molecular Diagnostics [61]. As others, we showed that the performance and spectrum of captured miRNAs is greatly affected by the library preparation protocol. In concordance with Godoy et al. [75], we documented a superior performance of EdgeSeq for accuracy, mapping rate and sensitivity, but it lacked in specificity. The most balanced performance was shown by randomized approach reaching well to average results in the majority of parameters. The similar conclusion was reached in the majority of other comparative studies (even though limited in the spectrum of compared methods) [58,59,60,75,76]. This emphasizes the robustness of the randomized protocol, which has been also recently selected by the Extracellular RNA Communication Consortium (ERCC) as a standard for their RNA-seq based studies [59,77]. Surprisingly, a good performance was achieved by QIAseq, although we were not able to confirm the beneficial effect of UMIs. Again, this is in concordance with the study of Herbert et al. [65], where QIAseq clustered with randomized and circularization approach that was the best rated in this comparison. Noteworthy, some studies performed using low input samples reported an even better QIAseq performance than achieved by using the randomized approach [36,78], suggesting the influence of template concentration. Although polyadenylation-based methods were shown to be more accurate than traditional ligation-based methods, their yield and overall mapping statistics are substantially lower [61,66]. A recent study showed that the proportion of mapped reads obtained using the Diagenode CATS protocol did not even reach units of percentage [66], even though such dramatic loss of reads was probably a consequence of the uniform mapping strategy used across all protocols in the study. Lastly, all the studies agreed on poor performance when using the traditional two-adaptor ligation protocols that introduce the largest proportion of bias that negatively affects most of the other metrics [36,60,76].

To sum up, the approach using randomized adaptors seems to be the most robust approach currently available. The status of this well-established method will probably even increase in the future, as some of the research consortia (ERRC) selected its application as a standard for small RNA-Seq studies [59,77]. For routine application, targeted approach EdgeSeq provides superior performance, although some protocol-specific negatives (lower specificity, expensive instrumentation) need to be considered before its employment.

**Table 2:** Overview of recently published benchmarking studies of small RNA-seq protocols. Some protocols were benchmarked when offered by different companies, therefore, the names may vary from the Table 1.

Benchmarking Study	Included Approaches	Commercial Protocols Included	Sample Types	Conclusion
Dard-Dascot, C. et al. BMC Genomics 19, 1–16 (2018) [58].	Original two-adaptor ligation	TruSeq Small RNA Library Prep Kit (Illumina) + in house modifications	Synthetic RNAs	Protocol utilizing randomized adaptors performed best
	Randomized adaptors	NEXTflex (Bioo Scientific) + in house modifications	*Arabidopsis thaliana* total RNA	on both human and plant miRNAs. Addition of polyethylene glycol (PEG)
	Polyadenylation and template switching	SMARTer (Clontech) and CATS (Diagenode)	Oilseed rape total RNA	and usage of chimeric DNA-RNA 5′ adaptor with random nucleotides
			HeLa cells total RNA	led to improved performance.
Coenen-Stass, A. M. L. et al. RNA Biol. 15, 1133–1145 (2018) [78].	Original two-adaptor ligation	NEBNext Multiplex Small RNA Library Prep Set (New England Biolabs)	Human plasma miRNAs	Protocols utilizing randomized adaptors and UMIs performed well across
	Randomized adaptors	NEXTflex (Bioo Scientific)	Human serum miRNAs	all measured characteristics and showed the least sequence bias.
	Polyadenylation and template switching	SMARTer (Clontech)	miRXplore universal reference	
	UMI	QIAseq miRNA library Kit (Qiagen)		
Giraldez, M. D. et al. Nat. Biotechnol. 36, 746–757 (2018) [59].	Original two-adaptor ligation	NEBNext Multiplex Small RNA Library Prep Set (New England Biolabs)	Human plasma total RNA	In-house adjusted protocol with randomized adaptors showed the least bias.
	Randomized adaptors	NEXTflex (Bioo Scientific) + in house modifications	Equimolar and ratiometric pool of synthetic RNAs	Optimization of ligation temperature and PEG helped to reduce bias.
Yeri, A. et al. BMC Genomics 19, 1–15 (2018) [76].	Original two-adaptor ligation	TruSeq Small RNA Library Prep Kit (Illumina)	Human brain total RNA	Protocol utilizing randomized adaptors detected the highest number of miRNAs
		NEBNext Multiplex Small RNA Library Prep Set (New England Biolabs)	Human liver total RNA	and had the highest correlation with results from ligation free methods, but
	Randomized adaptors	NEXTflex (Bioo Scientific) + in house modifications	Human placenta total RNA	it required user experience to be performed consistently.
	Sequencing of hybridization probes	EdgeSeq (HTG Molecular Diagnostics)	Human plasma total RNA	
	Hybridization-based technique	FirePlex (Abcam)		
Barberán-Soler, S. et al. Genome Biol. 19, 105 (2018) [25].	Original two-adaptor ligation	NEBNext Multiplex Small RNA Library Prep Set (New England Biolabs)	Human brain total RNA	Single adaptor ligation and circularization protocol detected the
	UMI	QIAseq miRNA library Kit (Qiagen)	miRXplore universal reference	the largest spectrum of miRNAs and showed the least bias.
	Randomized adaptors	NEXTflex (Bioo Scientific)		
	Polyadenylation and template switching	SMARTer smRNA-seq Kit (Takara Bio)		
	Single adaptor ligation and circularization	Beta version of RealSeq-AC Kit (Somagenics)		
Godoy, P. M. et al. Cell Rep. 29, 4212–4222.e5 (2019) [75].	Randomized adaptors	TruSeq Small RNA Library Prep Kit (Illumina) + in house modifications	Equimolar and ratio metric pool of synthetic RNAs	Small RNA-seq showed better specificity, ability to detect expected differential
	Sequencing of hybridization probes	EdgeSeq (HTG Molecular Diagnostics)	Human plasma total RNA	expression, but higher level of bias.
	Hybridization based techniques	FirePlex (Abcam) + nCounter (NanoString)		
Wong, R. K. Y. et al. BMC Genomics 20, 1–12 (2019) [36].	Original two-adaptor ligation	CleanTag Small RNA Library Prep Kit (TriLink BioTechnologies)	Human plasma total RNA	Protocol utilizing UMIs detected the highest number of miRNAs and correlated
	Randomized adaptors	NEXTflex (Bioo Scientific)		most closely to RT-qPCR validation data.
	UMI	QIAseq miRNA library Kit (Qiagen)		
Wright, C. et al. BMC Genomics 20, 513 (2019) [60].	Original two-adaptor ligation	TruSeq Small RNA Library Prep Kit (Illumina)	Human brain total RNA	Protocol utilizing randomized adaptors performed the best. Authors suggested
	Randomized adaptors	NEXTflex (Bioo Scientific)	miRXplore universal reference	usage of random nucleotides as UMIs to reduce PCR bias.
	Polyadenylation and template switching	SMARTer (Clontech)		
Heinicke, F. et al. RNA Biol. 17, 75–86 (2020) [66].	Original two-adaptor ligation	Small RNA-Seq Library Prep Kit (Lexogen) + TailorMix miRNA Sample Preparation Kit (SeqMatic)	Equimolar and ratio metric pool of synthetic RNAs	Best performance was shown by protocol utilizing UMIs. Polyadenylation
	CleanTag Small RNA Library Prep Kit (TriLink BioTechnologies)	Human CD8+ T cells total RNA	and circularization protocols showed a poor yield of miRNAs reads
	Single adaptor ligation and circularization	SMARTer microRNA-seq Kit (Takara Bio)		and were not considered for further analysis.
	Polyadenylation and template switching	CATS (Diagenode)		
	UMI	QIAseq miRNA library Kit (Qiagen)		
Herbert, Z. T. et al. J. Biomol. Tech. 31, 47–56 (2020) [65].	All available approaches to small RNA-seq	Small RNA-Seq Library Prep Kit (Lexogen) + TruSeq Small RNA Library Prep Kit (Illumina)	miRXplore universal reference	All methods showed high reproducibility and there was no protocol
		NEBNext Multiplex Small RNA Library Prep Set (New England Biolabs)	Mesenchymal stem cells total RNA	outperforming others across all the metrics.
		CleanTag Small RNA Library Prep Kit (TriLink BioTechnologies)		
		NEXTflex (PerkinElmer)		
		SMARTer smRNA-seq Kit + CATS (Diagenode)		
		RealSeq-AC Kit (Somagenics)		
		QIAseq miRNA library Kit (Qiagen)		
	Hybridization-based method	nCounter (NanoString)		
Baldrich, P. et al. bioRxiv (2020) [79]	Original two-adaptor ligation	NEBNext Multiplex Small RNA Library Prep Set (New England Biolabs)	Maize anthers total RNA	Protocols using randomized adaptors and two-adaptor ligation generated
		TruSeq Small RNA Library Prep Kit (Illumina)		highest number of reads mapping to miRNAs and phasiRNAs.
		CleanTag Small RNA Library Prep Kit (TriLink BioTechnologies)		
	Randomized adaptors	NEXTflex v2, NEXTflex v3 (Bioo Scientific)		
	Single adaptor ligation and circularization	RealSeq-AC Kit (Somagenics)		
	Polyadenylation and template switching	SMARTer smRNA-seq Kit (Takara Bio)		
Androvic, P. et al. bioRxiv (2021) [61].	All available approaches to small RNA-seq	Small RNA-Seq Library Prep Kit (Lexogen) + Small RNA Library Prep Kit (Norgen)	Human plasma total RNA	Protocols utilizing randomized adaptors and UMIs had the best overall
		QIAseq miRNA library Kit (Qiagen)	miRXplore universal reference	performance. Hybridization-based methods showed the highest sensitivity and
		NEXTflex (Bioo Scientific)		low specificity.
		SMARTer smRNA-seq Kit (Takara Bio)		
		RealSeq-Biofluids Kit (Somagenics)		
	Hybridization-based method	EdgeSeq (HTG Molecular Diagnostics)		

## 4. Considerations and Future Prospects

**Figure 4:**
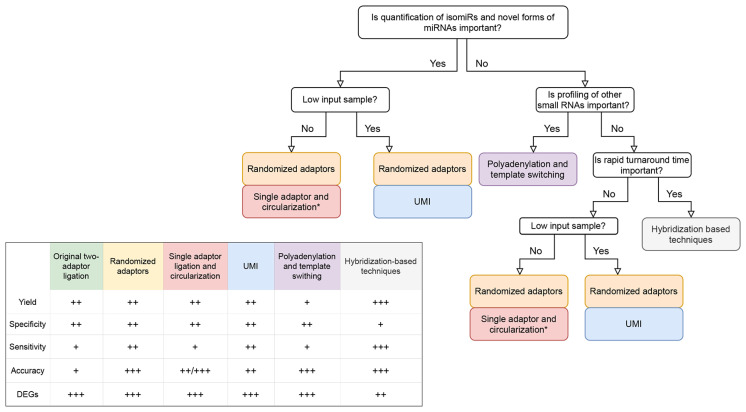
Selection of platform for small RNA-seq analysis. Decision diagram guides an informed choice of suitable protocol. Of note, other non-depicted experiment-specific criteria need to be further considered. Performance metrics represent a consensus of recent benchmarking studies listed in Table 2. +; poor, ++; average, +++; good, DEGs; Differential expression, *; Somagenics RealSeaq-AC Kit.

In the majority of comparative studies, none of the protocols outperformed others across all tested metrics. The results rather documented their variable performance and highlighted their different applications in miRNA research. When selecting a small RNA-seq protocol for the new study, the choice of the protocol should be therefore considered with respect to target-, sample-, and protocol-specific features, requirements on the analysis and expected outcomes. Here, we provide a simplified decision diagram for protocol selection (Figure 4) and summarize the basic aspects that need to be reflected.

(i) First, the choice of a protocol is given by the molecule of interest. miRNAs are enriched in the majority of ligation-based protocols, whereas other small RNAs along with miRNAs are captured by polyadenylation methods. As polyadenylation suffers from a low mapping rate and inability to distinguish isomiRs, protocol adjustments (specialized enzymes, ligation of cDNA) might be applied in the ligation-based protocols to capture also other small RNA classes [49,80].

(ii) Sample input requirement is another protocol-specific aspect for consideration. Although the majority of current methods supports low-input applications, including analysis of liquid biopsies, their performance varies broadly [59,61,75,76,78]. Using a plasma sample, protocols using randomized adaptors [59], alternatively UMIs (QIAseq specifically) [36] showed good performance. For routine analysis of crude biofluids sample, EdgeSeq platform outperformed other competitors [76]. More details on miRNA analysis in low-input samples might be found elsewhere [21,42].

(iii) After considering target- and sample-related factors, the accuracy of measurement is a key aspect in small RNA-seq analysis. As discussed, ligation and PCR biases might significantly confound the analysis and negatively influence the overall performance. To mitigate the bias while preserving good performance, hybridization-based approaches (EdgeSeq specifically) or randomized adaptors-based approaches are the methods of choice [61]. On rich RNA samples, the superior performance was documented for the circularization approach [25,65].

(iv) A common goal of small RNA-seq studies, is finding differential abundant miRNAs, the reproducibility of measurement needs to be considered as well. From this perspective, all available small RNA-seq methods demonstrated low within-protocol variability, allowing for precise differential expression analysis [75,76]. Although reproducible, one should keep in mind that in many cases the data suffered from strong protocol-specific bias, which limits any inter-protocol comparison.

(v) Lastly, the practical aspects of protocol selection are represented by the level of multiplexing, the complexity of library preparation or costs of measurement. The majority of protocols offers 96 indexes allowing the pooling of the same number of samples and their analysis in a single sequencing run. However, some protocols still offer a limiting level of multiplexing (48 or 24), which requests multiple sequencing runs and increases total costs in the case of analysis of large sample cohorts. From the view of the complexity of library preparation, EdgeSeq automated platform stands out over competitors, making the process of library preparation easy, fast and robust. This is however accompanied by expensive instrumentation and a higher cost of reagents.

In addition to existing protocols and platforms, there is ongoing research introducing novel improvements to small RNA-seq. When incorporated into current protocols, these advances possess a good promise to further increase the performance and applicability of small RNA-seq analysis. For example, the combination of optimized ligation temperature, the addition of polyethylene glycol (PEG) and 12bp-long randomized adaptors usable as UMIs have been demonstrated to achieve higher performance than commercially available NEXTflex [63]. Similarly, the use of 20% PEG during the ligation steps was shown to increase the sensitivity of the two-adaptor ligation protocol [81]. Another possible improvement is to use adaptors with randomized overhangs, which binds to the complementary ends of miRNAs. The utilization of this approach was shown to have less technical bias and significantly higher sensitivity than NEXTflex [82,83]. Moreover, it enables for profiling of small RNA species with 2′-O-methyl on 3′-end and therefore might be used for profiling of plant miRNAs [80]. Appealing is also the introduction of a higher level of multiplexing [84] that would allow for the analysis of large sample cohorts, employing the power and cost-effectiveness of modern sequencers. Apart from the improvement in wet-lab protocols, there is a possibility for the development of new computational methods for bias correction. The first step in this direction has been done in the study of Baroin-Tourancheau et al. [46], where authors reduced bias using a correction factor learned on the synthetic miRXplore standard. More advanced models, employing large sets of publicly available datasets or benchmarking studies could provide further progress in this field and finally allow such needed cross-study comparison. Lastly, we envision development in the data analysis pipelines. Currently, the miRNA analysis represents a complex process involving a large number of steps and analysis tools, with more than 1000 currently available [85,86]. This requires the assistance of experienced bioinformaticians and complicates the wide application of the technology. The development of user-friendly analysis platforms will help overcome this obstacle, promote standardization of the technology and its transfer to routine clinical practice.

## 5. Conclusions

Here, we reviewed the current state of small RNA-seq technology for analysis of miRNAs. Undoubtedly, small RNA-seq technology underwent a dramatic development in recent years. The major changes were targeted on the reduction of technical bias and simplification of the analysis workflow. The success rate of these efforts varies which is reflected by the diverse performance of small RNA-seq protocols in comparative studies. Overall, none of the protocols are superior to others and experiment-specific factors need to be carefully considered when selecting a protocol for a new study. To guide the selection, we summarized all major advancements in the field and provided our perspective on their applicability for miRNA profiling. We believe that small RNA-seq holds the promise to become a leading technology for the analysis of miRNAs and once standardized, its applicability will grow even more in popularity.

## Figures and Tables

**Table 1:** Overview of commercially available protocols for small RNA-seq analysis.

Technology	Product Name	Company	Reference
Original two-adaptor ligation	Small RNA-Seq Library Prep Kit	Lexogen GmbH, Vienna, Austria,	not available
	Small RNA Library Prep Kit	Norgen Biotek Corp., Thorold, ON, Canada	not available
	TruSeq Small RNA Library Prep Kit	Illumina, San Diego, CA, USA	not available
	TailorMix miRNA Sample Preparation Kit	SeqMatic, Fremont, CA, USA	not available
	NEBNext Multiplex Small RNA Library Prep Set	New England Biolabs, Ipswich, MA, USA	not available
	CleanTag Small RNA Library Prep Kit	TriLink BioTechnologies, Inc., San Diego, CA, USA	[22]
	ScriptMiner Library preparation Technology for Small RNA	Cambio Ltd., Cambridge, UK	[23]
Randomized adaptors	NEXTflex Small RNA Sequencing Kit	PerkinElmer, Waltham, MA, USA	[24]
Single adaptor ligation and circularization	RealSeq-AC Kit	Somagenics, Santa Cruz, CA, USA	[25]
	RealSeq-biofluids Kit	Somagenics, Santa Cruz, CA, USA	not available
	SMARTer microRNA-Seq Kit	Takara Bio, Shiga, Japan	not available
UMI	TrueQuant SmallRNA Seq Kit for Ultra Low Input	GenXPro GmbH, Frankfurt Main, Germany	not available
	QIAseq miRNA Library Kit (12bp UMIs)	Qiagen, Hilden, Germany	not available
Polyadenylation and template switching	SMARTer smRNA-seq Kit	Takara Bio, Shiga, Japan	not available
	CATS Small RNA-seq Kit	Diagenode, Liege, Belgium	not available
Sequencing of hybridization probes	HTG EdgeSeq miRNA Whole Transcriptome Assay	HTG Molecular Diagnostics, Inc., Tuscon, AZ, USA	not available
Hybridization based techniques without NGS readout	FirePlex miRNA assays	Abcam, Cambridge, UK	not available
	nCounter miRNA Expression Panels	NanoString, Seattle, WA, USA	[26]
